# The Consumption of Cannabis by Fibromyalgia Patients in Israel

**DOI:** 10.1155/2018/7829427

**Published:** 2018-07-22

**Authors:** George Habib, Irit Avisar

**Affiliations:** ^1^Rheumatology Unit, Laniado Hospital and Rheumatology Clinic, Nazareth Hospital and Faculty of Medicine, Technion-Israel Institute of Technology, Haifa, Israel; ^2^Cann Pharmaceutical, Israel

## Abstract

**Objective:**

To report on the habits of cannabis consumption among fibromyalgia patients in Israel.

**Patients and Methods:**

An Internet-based questionnaire was posted to three large fibromyalgia Facebook groups in our country. The questionnaire was anonymous and included demographic, clinical, and cannabis-related questions, including acquisition of a license for medical cannabis (MC) method and amount of cannabis consumption; need to buy cannabis beyond the medical allowance; effect of cannabis on pain, sleep, depression, and anxiety; adverse effects of cannabis; feelings of dependence on cannabis or other meds; the involvement of family members; tendency to drive after using cannabis; and employment and social disability status.

**Results:**

Of 2,705 people, 383 (14%) responded to the questionnaire, with a mean age of 42.2±14.2 years. Of the responders, 84% reported consuming cannabis, and 44% were licensed for MC. The mean amount per month of cannabis consumed was 31.4±16.3g, and 80% of cannabis consumers (CC) smoked pure cannabis or cannabis mixed with tobacco. Pain relief was reported by 94% of CC, while 93% reported improved sleep quality, 87% reported improvement in depression, and 62% reported improvement in anxiety. Of MC-licensed CC, 55% bought cannabis beyond the medical allowance on the black market. Adverse effects were reported by 12% of CC. Only 8% reported dependence on cannabis. Most CC (64%) worked either full- or part-time jobs, and 74% reported driving “as usual” under cannabis use.

**Conclusions:**

Cannabis consumption among fibromyalgia patients in our country is very common and is mostly not licensed. Nearly all CC reported favorable effects on pain and sleep, and few reported adverse effects or feeling of dependence on cannabis.

## 1. Introduction

Fibromyalgia is a chronic pain syndrome characterized primarily by diffuse musculoskeletal pain, fatigue, and mood and sleep disturbances [1]. It affects women more frequently than men and has a genetic preponderance [2]. It is not uncommon, as its prevalence in the general population is estimated around 7% [3].

Fibromyalgia might have a tremendous physical and psychological impact on patients and may lead to disability [4]. Currently, there is no cure for this syndrome in most patients, and the main treatment is usually pain control medication. These medications include pregabalin and duloxetine [5]. Some patients may benefit from benzodiazepines, tricyclic antidepressants, and other antidepressants [6]. Many of these medications are associated with adverse effects that affect compliance.

Cannabis is considered an illicit drug in most countries, including our country. However, it is widely used in both illegal and legal fashions [7]. In legal forms, it is used primarily by cancer patients. Many of its users report the high potential of cannabis to suppress pain and induce sleep and calmness [8]. Due to these favorable properties, cannabis has been legislated during the last few years, in some states in the United States and other countries for medical use [9, 10]. In our country, medical cannabis (MC) is licensed by the Medical Cannabis Agency (MCA), a special committee at the Ministry of Health, for specific indications, including uncontrolled pain in cancer patients, uncontrolled gastrointestinal symptoms in Crohn's patients, uncontrolled seizures, uncontrolled Parkinson's disease, posttraumatic stress disorder (PTSD), unresponsive diabetic neuropathy, and other indications such as degenerative or inflammatory musculoskeletal problems. The application for MC licensing is sent directly to the MCA by the subspecialty physician. Fibromyalgia is not considered as one of these indications, based on the recommendation of the Rheumatology Association of our country. However, fibromyalgia patients who are followed also at other subspecialties such as pain clinic and/or gastroenterology (GI) clinic for GI symptoms and/or followed by psychiatric for mood disturbance might get approval for MC. While many are not approved for MC, many patients with fibromyalgia in our country have experience using medical or nonmedical cannabis.

There are no studies about the prevalence of cannabis consumption by fibromyalgia patients in our country. There are very few studies in the literature about the use of cannabis by fibromyalgia patients from other parts of the world [11, 12]. In this study, we report on the habits of cannabis consumption of three large fibromyalgia social media groups in our country.

## 2. Materials and Method

Members of three large Facebook groups in our country with fibromyalgia diagnosed by a rheumatologist were asked to fill out an anonymous questionnaire online. The questionnaire included demographic, clinical, and cannabis-related parameters, including age; social status; duration of fibromyalgia; current treatment; amount and method of cannabis consumption (smoking, vaporization, oil); acquisition of a license for MC; prior application for MC; sufficiency of the legal allowing of MC; effect of cannabis on pain, sleep, anxiety, and depression; feelings of addiction to cannabis; feelings of addiction to other meds; tendency to drive a motor vehicle under cannabis treatment; employment status; and impact of cannabis on employment and social security disability.

This study was approved by the local ethics committee of our hospital, and all participants agreed (anonymously) through the net to fill out the questionnaire as a part of research.

### 2.1. Statistical Analysis

For statistical analysis, simple frequency measures were used using Excel Microsoft software.

## 3. Results

Out of the fibromyalgia social media groups' 2,705 members, 383 participants (~14%) with fibromyalgia diagnosed by a rheumatologist responded to the questionnaire.

Table 1 summarizes the demographics of the patients. Of these, 85% were female with a mean duration of fibromyalgia of 8.26±7.8 years.

Table 2 summarizes the data regarding cannabis consumption and related issues. Approximately 84% of the participants reported consuming cannabis with a mean dose of 31.4±16.3g per month. Only around 12% of cannabis consumers (CC) reported adverse effects, and approximately 92% denied feeling addicted to cannabis.

Table 3 summarizes the medical and nonmedical impact of cannabis. Participants reported pain improvement and better quality of sleep under cannabis consumption at 94% and 93%, respectively. Part- or full-time jobs were held by 64% of the patients under cannabis consumption. Around 72% of the patients continued to drive while under cannabis treatment. Most of the patients did not file a request for social security disability; of those who did apply for social disability, the mean disability was ~51%.

## 4. Discussion

There were many interesting findings in our study. The main finding was that nearly 84% of those who responded to the questionnaire were CC. This rate is very high, and it raises the question of whether the responders of the questionnaire represent the whole community of the three fibromyalgia Facebook groups. A response rate of 14% to an Internet questionnaire is considered an accepted rate [13, 14], and the correlation between a paper-based survey and electronic survey was high in one study [13]. This may actually indicate that more than 80% of the total number (2,705) of patients in the fibromyalgia social media groups were consuming cannabis.

The rate of approval for MC licensing by the MCA was also relatively high (142/317, 45%) among the fibromyalgia participants in our study. However, it must be remembered that many of these patients likely had concurrent medical conditions that facilitated or were the main reason for approval. Unfortunately, the questionnaire did not include questions regarding specific diseases or conditions such as malignancy that the patients might also have, in the interest of improving compliance with the questionnaire. Licenses for MC use were issued by the MCA after an appeal in about 37% of the MC-licensed patients; among some, three appeals were required prior to approval. Therefore, appealing for MC licensing after refusal by the MCA is always recommended.

The mean quantity of cannabis consumed by all CC was nearly 1g per day, and nearly two-thirds of the patients licensed for MC reported that their supplied quantity was not sufficient. More than half (~55%) reported buying cannabis from the black market.

It must be remembered that the price of the supplied MC for licensed patients is fixed regardless of the quantity supplied, standing at about $120 a month, while those buying on the black market sometimes pay as high as $1,450 a month to get cannabis, figures that are quite high. These figures reflect the high demand for cannabis by fibromyalgia patients in our country.

Most of MC consumers used three or more species of cannabis. This indicates that one species of cannabis cannot suffice for the different complaints of the patients, such as pain, insomnia, anxiety, or lack of energy. Different species and different concentrations of tetrahydrocannabinol (THC) and cannabidiol (CBD) were needed to tackle these complaints. Unfortunately, we did not specifically document the concentration of THC or CBD in each species.

Most of the patients used unlicensed cannabis by smoking it mixed with tobacco, while those using MC smoked it without mixing it. Some used vaporization and some used oral oil drops. Vaporization appealed primarily to women who did not want to smoke, while cannabis oil drops were considered weak in terms of efficacy. Oil drops, however, had a long term effect and were usually used as a complement to smoking or vaporization.** The other advantage of oil drop preparations of cannabis is the accurate measurement of both THC and CBD that patients eventually get**.

As expected, a high percentage of patients (94%) reported pain relief; the impact on sleep, a major problem among fibromyalgia patients, was similar, making cannabis a versatile remedy. It also improved depression and anxiety, though in a lower percentage of patients. All these effects make cannabis very appealing to fibromyalgia patients.

Only 12% of all cannabis users in our study reported adverse effects, compared to 94% reporting adverse effects from other pain meds prior to cannabis use. Most cannabis-related adverse effects were mild and transient such as eye or throat irritation (data not shown). In addition, only 8% of cannabis users felt dependent on cannabis, as opposed to 66% feeling dependent on other pain meds. On the other hand, nearly 85% of the patients either completely stopped taking any other pain medications or reduced the dosage of other meds. This reflects the advantage of cannabis over other meds in alleviating pain in addition to its favorable effects on sleep and mood.

Eighty-one percent (81%) of the patients reported an improvement in their capacity to perform daily activities, and 64% reported going back to part- or even full-time jobs. These figures are very important to the patients personally, to their families, and to society in general. We could not find data in the literature about this issue among patients treated with cannabis for different indications. However, in a large study assessing the impact of cannabis as an illicit drug, a detrimental effect was found on employment and labor force [15].

Although the MCA explicitly explains that it is forbidden to drive under cannabis use, most patients do drive under the effect of cannabis, with only about 21% of licensed patients not driving at all under cannabis treatment. It could be that those patients who drive under the influence of cannabis feel focused enough to proceed with driving.

Although fibromyalgia is not listed among the medical conditions for social security liability, there is an increasing awareness by social security employees, and more and more patients are approved for disability benefits, although the percent of disability granted for these patients is still lower than the real physical and functional disabilities they face. Still, many patients are granted no social security disability, and the 51% figure in our study actually reflects total disability, mainly due to these patients' other diseases and medical conditions.

Eighty-nine percent (89%) of the patients do not keep their use of cannabis a secret but share this information with their close family members. This fact probably reflects the favorable social effect of cannabis enabling patients to spend more time with their family members rather than seeking isolation and the increased general awareness of this type of treatment in the country.

The results of our study should encourage both the Rheumatology Association in our country and the MCA to reconsider their stand on cannabis and include fibromyalgia among the indications for MC under certain restrictions.

## Figures and Tables

**Table 1:** Demographics of all participants with fibromyalgia who responded to the questionnaire (383 participants).

Parameter	Results (%)
(i) Number of females	325 (85)
(ii) Age (yrs)	42.2±14.2, 19-74*∗*
(iii) Duration of fibromyalgia (yrs)	8.26±7.8, 1-40*∗*
(iv) Social status	
Married	238(62)
Divorced	57 (15)
Single	85 (22)
Widow	4 (1)
(v) Number of kids	1.81±1.42, 0-4*∗*

*∗* mean± SD, range.

**Table 2:** Cannabis and other treatment related parameters of all participants.

Parameter	Results (%)
(i) Total number of patients who uses cannabis	323/383 (84)
(ii) No. of patients licensed for MC	142/323 (44)
(iii) No. of patients who consume cannabis without a license	181/323 (56)
(iv) No. of patients who applied for MC license	317/383 (83)
(v) No. of patients who appealed after refusal	65
(vi) No. of appeals prior to licensing	
1	27
2	16
3	10
(vii) No. of licensed patients reporting MC supplied not enough	94/142 (66)
(viii) No. of licensed patients who buy in black market	78/142 (55)
(ix) Amount of cannabis consumed/ month (g) by all the patients	31.4±16.3, 5-150*∗*
(x) Amount of money paid for cannabis in the black market (US$)	106±206, 29-1428*∗*
(xi) Frequency of cannabis consumption/day	4.7±3.96, 1-20
(xii) No. of patients using ≥ 3 species of MC	63/142 (~44)
(xiii) No. of patients using 2 species of MC	50/142 (~35)
(xiv) No. of patients using 1 species of MC	29/142 (~21)
(xv) Method of cannabis consumption by all participants	
Smoking with cigarette	241/323 (63)
Smoking pure cannabis	85 (~17)
Oil under tongue	18 (~5)
Vaporizing	57 (~15)
(xvi) No. of patients reporting adverse effects from cannabis	36/308 (12)
(xvii) No. of patients reporting feeling dependent on cannabis	25/304 (8)
(xviii) No. of patients reporting adverse effects from regular meds for fibromyalgia prior to cannabis use	336/357 (94)
(xix) No. of patients reporting dependence on regular meds	229/348 (66)
(xx) Patients' report on the use of other meds under cannabis	
Using the same other meds	45/304 (15)
Sopped all other meds	134/304 (44)
Consuming less other meds	125/304 (41)

**∗** mean±SD, range.

No. = Number.

**Table 3:** Impact of cannabis on medical and nonmedical parameters.

Parameter	Results (%)
(i) No. of patients reporting pain relief due to cannabis	303/323 (94)
(ii) No. of patients reporting better quality of sleep	281/302 (93)
(iii) No. of patients reporting improvement of depression	172/202 (85)
(iv) No. of patients reporting improvement of anxiety	122/235 (62)
(v) No. of patients reporting improving of daily activity	237/291 (81)
(vi) Patients reporting sports activity prior to cannabis	89/309 (32)
(vii) Patients reporting sport activity under cannabis	129/301 (43)
(viii) Patients report on work	
Working full-time job under cannabis	152/323(47)
Partial time	55/323 (17)
Not working at all	116/323 (36)
(ix) No. of patients driving under cannabis	
As usual	233/323 (72)
Not at all	29/323 (9)
I don't drive at all	61/323 (19)
(x) No. of patients with social security disability	146/374 (39)
(xi) Percent of disability	51.3±29.7, 0-100*∗*
(xii) No. of patients who informed family members about cannabis use	
Yes	214/323 (66)
Partially	74/323 (23)

*∗* mean± SD, range.

No. = number.

## Data Availability

All data used in this study is shown in the Results.
